# First detection and complete genome sequence of a new potexvirus naturally infecting *Adenium obesum*

**DOI:** 10.1007/s00705-023-05871-x

**Published:** 2023-09-07

**Authors:** Marie-Emilie A. Gauthier, Shamila W. Abeynayake, Ruvini V. Lelwala, Cassie A. McMaster, Robin Eichner, Jennifer Morrison, Candace E. Elliott, Sonia Fiorito, Adrian Dinsdale, Julie Pattemore, Roberto A. Barrero

**Affiliations:** 1 eResearch, Research Infrastructure, Academic Division, Queensland University of Technology, Brisbane, QLD 4000 Australia; 2 Department of Agriculture, Forestry and Fisheries, PO Box 858, Canberra, ACT 2061 Australia; 3 Department of Animal, Plant and Soil Sciences, Centre for AgriBiosciences, La Trobe University, Bundoora, VIC 3086 Australia

## Abstract

Here, we report the detection and complete genome sequence of a novel potexvirus, tentatively named “Adenium obesum virus X” (AobVX), isolated from *Adenium obesum*, that was sent for virus screening at Australian Government post-entry quarantine (PEQ) facilities after being imported into Australia from China. The AobVX genome is 6781 nucleotides in length excluding the poly(A) tail and is predicted to encode conserved potexvirus proteins and sequence motifs across five open reading frames. The RNA-dependent RNA polymerase of this virus shares the highest amino acid sequence similarity with that of nerine potexvirus 1 (58.7% identity) and nerine virus X (58.58% identity). This is the first report of a positive-sense single-stranded RNA virus in *A. obesum* related to members of the genus *Potexvirus* in the family *Alphaflexiviridae.*

**Supplementary Information:**

The online version contains supplementary material available at 10.1007/s00705-023-05871-x.

*Adenium obesum* (*Forssk.*) *Roem.* and *Schult.* (family Apocynaceae), commonly known as desert rose, is a perennial succulent shrub native to southeast Africa. It is a popular exotic ornamental that is gaining prominence due to its drought resistance, ease of maintenance, and the variety of colours and shapes of its abundant and long-lasting flowers [1]. It is a known host to some viruses, such as cucumber mosaic virus [2] and tomato spotted wilt virus [3], but not potexviruses. Potexviruses are characterized by non-enveloped flexuous filaments (470–580 × 13 nm) that contain a monopartite single-stranded RNA genome ranging between 5.9 and 7.0 kb in size. A typical potexvirus genome is composed of a 5’ cap structure, five open reading frames (ORFs), and a 3’ poly(A) tail. It encodes an RNA-dependent RNA polymerase (RdRp), triple gene block (TGB 1, 2, and 3) proteins, and a coat protein (CP). Potexviruses mainly infect herbaceous hosts and have no known vectors [4].

In November 2021, mottling symptoms were observed on leaves of a batch of imported *A. obesum*, which were growing in an approved arrangement at PEQ Nambour Queensland (Fig. 1A). The plants were tested by the Department of Agriculture, Forestry and Fisheries, using ImmunoStrip testing (Agdia), and were positive for cucumber mosaic virus (CMV). Transmission electron microscopy examination revealed that the sap from the infected plants also contained filamentous particles approximately 500 nm in length and 11–20 nm in diameter (Fig. 1B), morphologically similar to potexviruses.

**Fig. 1:**
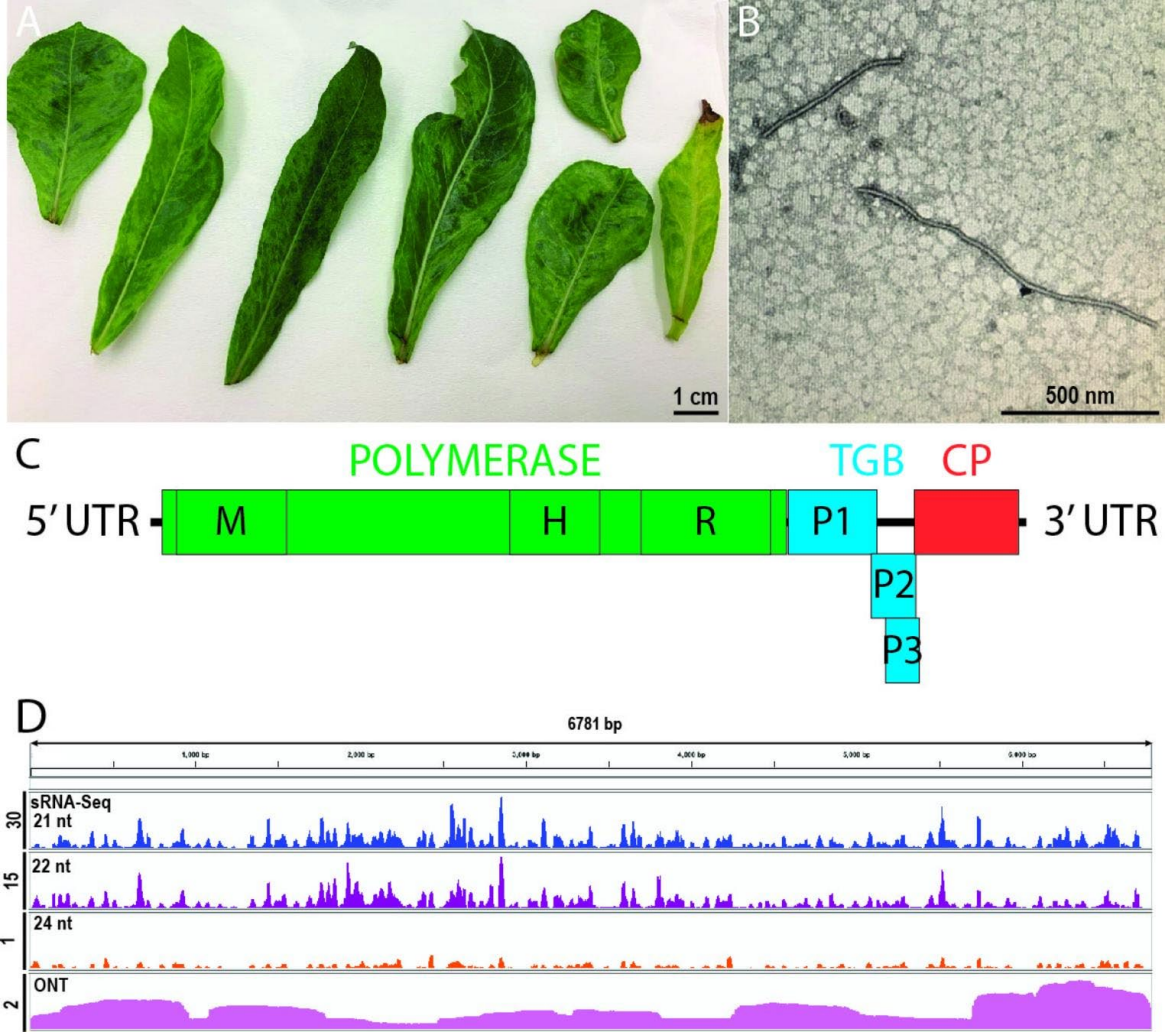
(A) *Adenium obesum* leaves infected with AobVX and CMV showing mottling symptoms. (B) Morphology of AobVX virions, viewed by transmission electron microscopy. Samples were prepared as per Le Blanc et al. [5]. (C) Schematic representation of the genome organization of AobVX. The 5′ and 3′ UTRs are represented by solid horizontal bars, while the ORFs are depicted as open boxes. CP, coat protein; H, helicase domain; M, methyltransferase domain; R, RdRp domain; TGB, triple gene block; P1, TGB protein 1; P2, TGB protein 2; P3, TGB protein 3. (D) Aligment of small RNAs and Oxford Nanopore Technology (ONT) reads derived for sample MT483 onto the AobVX genome sequence. Scale bars on the *y*-axis show the total number of mapped reads (×1000).

Total RNA was extracted from a symptomatic plant, and a small RNA (sRNA)-Seq library was generated and sequenced as recommended by Lelwala et al. [6]. *De novo* assembly of the quality-filtered reads obtained followed by contig scaffolding using VirReport [7] enabled the detection of CMV, as well as the full genome reconstruction of a novel potexvirus. Mapping of sRNA reads onto the AobVX genome sequence resulted in 1,658,356 aligned reads (mean read depth, 244), of which 62.7%, 29.3%, and 0.6% were 21 nt, 22 nt, and 24 nt long, respectively (Fig. 1C). The high proportions of mapped 21-nt and 22-nt reads were consistent with them being derived from the plant antiviral RNAi silencing response [8]. The AobVX genome was recovered from seven additional *Adenium* plants showing similar symptoms. The nucleotide sequence has been deposited in the GenBank database under the accession number OR039325, and the raw data are available under BioProject PRJNA984418 (reviewer link available at https://dataview.ncbi.nlm.nih.gov/object/PRJNA984418?reviewer=vfrple83dh9oq2f6o04eieohhm).

The complete sequence of the sRNA-based genome assembly, including the 5’ and 3’ untranslated regions (UTRs), was independently confirmed in one of the *Adenium* specimens tested by coupling rapid amplification of cDNA ends (RACE) [9] and Oxford Nanopore Technology (ONT)-based rapid amplicon sequencing (Supplementary Table S1) as described previously [10]. The scaffold obtained was > 99% identical to the original sRNA-based genome assembly. Mapping of the ONT reads to the AobVX genome sequence using minimap2 [11] resulted in 6501 aligned reads (mean read depth, 235) (Fig. 1C).

The genome sequence of AobVX is 6781 nt long and predicted to contain five ORFs, which are flanked by a 91-nt 5’ UTR and a 50-nt 3’ UTR (Fig. 1D). The 5’ UTR begins with the conserved motif “GGAAAA” [12], and the 3’ UTR harbours the conserved hexamer “ACUUAA” [13]. The first and second ORFs are separated by a 12-nt-long intergenic region, while the last four ORFs (2–5) overlap slightly with one another. This genomic organization has been reported in other potexvirus members [14].

ORF1 (4833 nt) encodes an RdRp of 1610 aa that is predicted to contain the three conserved replicase domains of the “alphavirus-like” supergroup of RNA viruses: (a) an N-terminal putative methyltransferase domain, (b) a helicase domain beginning with the NTP-binding consensus motif “GX_2_GXGKS”, (c) and a C-terminal RdRp domain. The region between the putative methyltransferase and the helicase domains shows the most variation both in terms of length and sequence similarity, as has been observed in other potexviruses [15]. ORF2 (687 nt), ORF3 (344 nt), and ORF4 (261 nt) compose the TGB structure and encode polypeptides of 228 aa (TGB1), 114 aa (TGB2), and 86 aa (TGB3), respectively. TGB1 is predicted to also contain an NTPase/ helicase domain. ORF5 (807 nt) encodes a 268-aa CP, which contains the conserved hydrophobic sequence “FAAFDFFDAV” within the predicted coat protein domain (Fig. 1D, Supplementary Fig. S1).

**Fig. 2:**
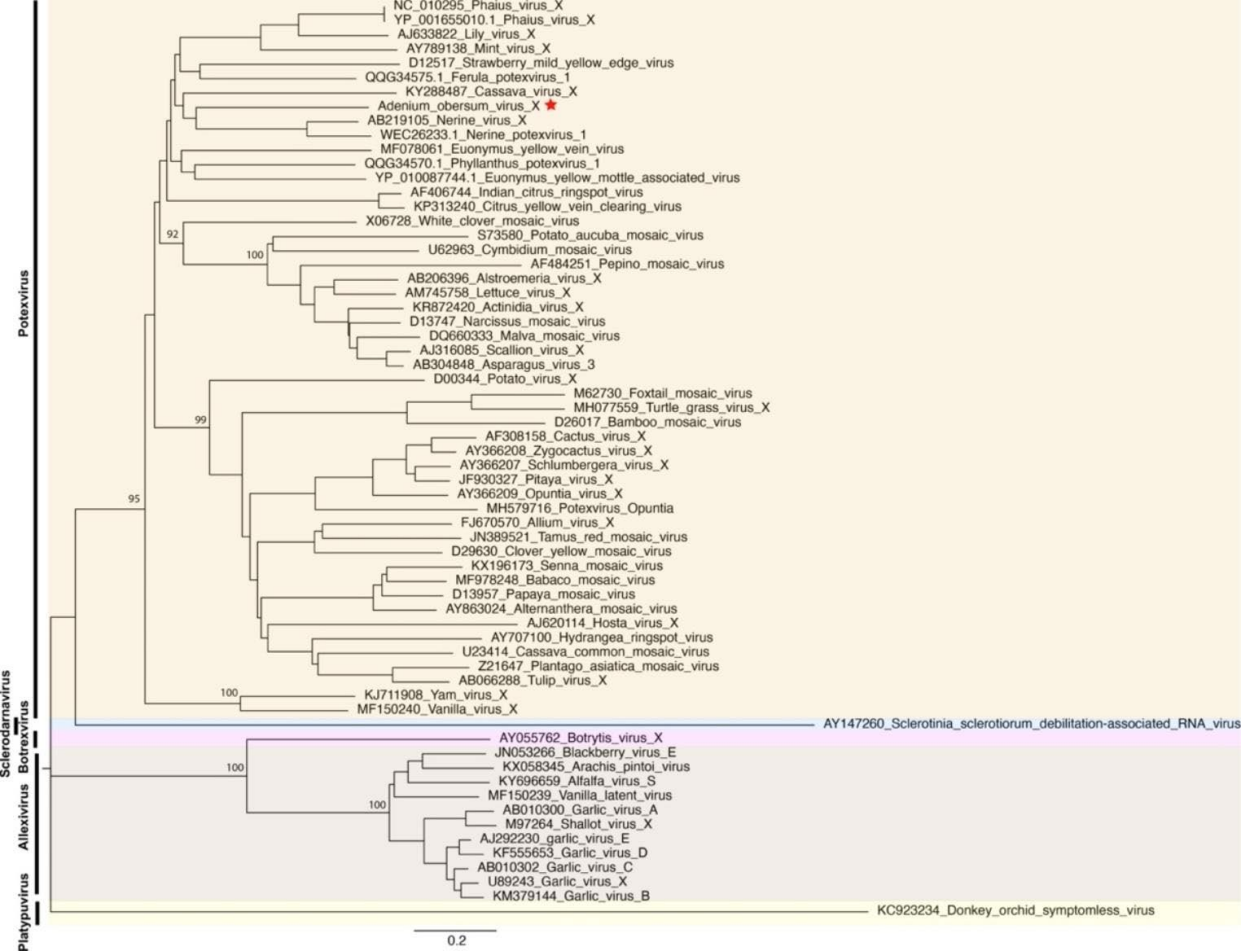
Neighbour-joining phylogenetic tree based on the predicted replicase amino acid sequence of AobVX and 63 members of the family *Alphaflexiviridae*, using 1000 bootstrap replicates. Analysis was performed using MEGA X. Percent boostrap values > 50 are shown at each major node.

Pairwise comparisons indicated that the predicted RdRP of AboVX showed the highest amino acid sequence similarity to that of nerine potexvirus 1 (58.7%) and nerine virus X (58.58%) (Supplementary Table S2). This is consistent with the results of phylogenetic analysis based on the RdRp sequences of AobVX and those of other representatives of the family *Alphaflexiviridae*, in which AobVX clusters with potexviruses and is most closely related to nerine potexvirus 1 and nerine virus X (Fig. 2). Based on its morphology, genome organization, sequence similarity, and phylogenetic clustering with previously reported potexviruses, the newly isolated virus AobVX is proposed to be a member of a distinct species in the genus *Potexvirus*.

### Electronic Supplementary Material

Below is the link to the electronic supplementary material

Supplementary Material 1
