# Supplementary material for: First detection and complete genome sequence of a new potexvirus naturally infecting Adenium obesum
Source: Arch Virol. 2023 Sep 7;168(10):244. doi: 10.1007/s00705-023-05871-x (PMC10484807; doi:10.1007/s00705-023-05871-x)
Supplement: Supplementary file 1 — Supplementary Material 1 [file 705_2023_5871_MOESM1_ESM.docx]

Title: First detection and complete genome sequence of a new potexvirus naturally infecting *Adenium obesum.* Journal*:* Archives of Viology

Authors: Marie‐Emilie A Gauthier, Shamila Weerakoon Abeyanake, Ruvini V. Lelwala, Cassie McMaster, Robin Eichner, Jennifer Morrison, Candace E. Elliott, Sonia Fiorito, Adrian Dinsdale, Julie Pattemore & Roberto A. Barrero

**Affiliation and email address of corresponding author**: eResearch, Research Infrastructure, Academic Division, Queensland University of Technology, Brisbane, QLD, 4000, Australia. roberto.barrero@qut.edu.au

**Supplementary Table 1.** Polymerase chain reaction primers used for the amplification of the AobVX genome.

|  | **Primer**  **name** | **Nucleotide sequence 5’-3’** | **Position (nt)** | **Product size (nt)** |
| --- | --- | --- | --- | --- |
| Amplicon PCR R8 for ONT (Rapid) | Potex-P2F1 | ACGACCCATCCTCAAACAAG | 175-194 | 1623 |
|  | Potex-P1R1 | TAGCATTGCACCTTGAGCAC | 1778-1797 |  |
| Amplicon PCR R9 for ONT (Rapid) | Potex-P1F1 | GCCAATTTCTCCCAAGATGA | 1070-1089 | 2133 |
|  | Potex-P3R1 | GCCCAGCTTATTTGCAATGT | 3183-3202 |  |
| Amplicon PCR R10 for ONT (Rapid) | Potex-P3F1 | TCAAAGGCAATCAGATGCAG | 2457-2476 | 1382 |
|  | Potex-P5R1 | TGCCAGCTCTTTCAGGTTTT | 3819-3838 |  |
| Amplicon PCR R11 for ONT (Rapid) | Potex-P5F1 | TTACCCCCTCCATGATCAAA | 3273-3292 | 1760 |
|  | Potex-P6R1 | GCCTGCTACCAGGTGGATTA | 5013-5032 |  |
| Amplicon PCR R12 for ONT (Rapid) | Potex-P4F1 | GACCTCAACCAATTCGTGCT | 4232-4251 | 2226 |
|  | Potex-P7R1 | GGTGGTGATTGCCTCTCAAT | 6438-6457 |  |
| Amplicon PCR 5’end for ONT (Ligation) | Forward universal | AAGCAGTGGTATCAACGCAGAG | NA | 980 |
|  | Potex-P2R1 | CATGGCAAGAGTTTTGCTGA | 960-979 |  |
| Amplicon PCR 3’end for ONT (Ligation) | Potex-P7F1 | TCTACCCCACACTGGTGACA | 5682-5701 | 1100 |
|  | 3’ RACE | GGCCACGCGTCGACTAGTAC | NA |  |

**Supplementary Table 2.** Percentage amino acid identity of the RNA-dependent RNA polymerase, triple gene block proteins and coat protein of *Adenium obesum* virus X to the most closely related viruses in *Potexvirus*. AobVX meets the criterium by the International Committee on Taxonomy of Viruses to qualify for a new species of *Potexvirus* (< 80% aa identity between their RdRp and CP genes) [4].

| **Virus** | **ORF1 (RdRP)** | **ORF2 (TGBp1)** | **ORF3 (TGBp2)** | **ORF4 (TGBp3)** | **ORF5 (CP)** |
| --- | --- | --- | --- | --- | --- |
| Cassava virus X (NC_034375.1) | 55.11 | 35.32 | 25.69 | NA | 43.65 |
| Citrus yellow vein clearing virus (MW429491.1) | 44.38 | 33.33 | 38.6 | 37.04 | 37.50 |
| Nerine potexvirus 1 (MZ643995.1) | 58.70 | 37.50 | 42.73 | 46.88 | 40.89 |
| Nerine virus X (NC_007679.1) | 58.58 | 34.68 | 43.12 | 28.57 | 40.82 |
| Phyllantus potexvirus 1 (MW328725.1) | 57.44 | 32.16 | 41.44 | 42.42 | 41.44 |
| Yam virus X (NC_025252.1) | \| 53.70 \|  \| \| --- \| --- \| | 29.6 | 49.4 | 32.43 | 44.93 |

**PF00286** 61 EALAAVIKES CTLRQFCAYYAKVVWNHMLTHNTPPANWAAKGFTEETK**FAAFDFFDAV**LSPAALP PPE 125 Flexi_CP

OR039325.1 144 DTLARITKKH TTLRKFCSYYAKIVWNLLIERQSPPAGWAKWNYRKSEAFAAFDFFDAVTNPAALE PKE 210 AobVX

ARG47560.1 94 STLAGAVKSV TTMRRFCSYYAKVVWNMLLRENTPPASWAKMGFIEEAKFAAFDFFDAVRSPAALE PKG 161 CsVX

QDX18380.1 276 MEVAAIIKKH TTLRRFCGFYAKIVWNIMLVTNIPPSGWMKKGYKENTKFAAFDFFVHVSNNAALE PEN 343 APV1

ABY53444.1 126 QTLAGLVKTV CTLRQFCMFYAKVVWNMMLKTEKPPASWQKWEYRYTERYAAFDFFQGVSNEAALN PTD 201 AAV1

6R7G_F 83 ARLAAAIKEV CTLRQFCMKYAPVVWNWMLTNNSPPANWQAQGFKPEHKFAAFDFFNGVTNPAAIM PKE 150 PVX

Q04572.1 140 ADLKTLWKAS ATLRQFCSYYAKSCYVSGKQQKKPPANWSRKGYPEEAKFAGFDFFNAVLSESSPA PPG 207 [ShVX](https://www.ncbi.nlm.nih.gov/Taxonomy/Browser/wwwtax.cgi?id=31770)

B1PS80.1 173 EAAVGVIKEI LTLRQFAAYYATFVWNWGIKNEIPPANWVAKGYTDETKYAAFDTFSYVGSPLGLR.[1].TPT 241 LoLV

Q9QEE7.1 203 NALAGVVRDF CPLRAFCAYYSRVVWNLMIKADQPPANWMKSGIDEGAKFAAFDFFHGVLSPASLY.[1].PLE 271 ICRSV

P17529.2 179 DAVLAVLKKD.[1].ETLRRVCRLYAPVTWNHMLTHNAPPADWAAMGFQYEDRFAAFDCFDYVENTAAVQ PLE 247 PVM

Q64966.1 291 EEVGTMIKQT.[2].CTLRQYCAFYAKHVWNLMLQTQSPPANWVGKEFKFETRYAAFDFFFGVESTASLE PAD 360 ASPV

Q6YNQ5.1 277 ELIAAHVKEH CTIRQFCSYFAKVVWNHLLTHATPPVNWAKHGFTLDSRYAAFDFFDAVTNAAALP PKN 344 BVX

Q91QZ1.1 235 KDIAYAIRTS.[1].ITVRQFCAAFANLYWNFNLARNTPPENWRKKGFTEGTKFAAFDFFYAVGSNAAIP.[1].EAD 304 CLBV

**Supplementary Figure 1.** Amino acid alignment of *Adenium obesum* virus X and a selection of representatives from the order *Tymovirales*, showing the hydrophobic motif “FAAFDFFDAV” located within the 3’ end of the predicted consensus superfamily viral coat protein domain Flexi_CP (PFOO286). The motif is displayed in bold. Figure adapted from the output obtained using the NCBI conserved domain CD-Search tool. Highly conserved amino acids are displayed in red and less conserved domains in blue, using default colour bits threshold of 2.0. The number of unaligned residues is indicated in grey in brackets. **AAV1** Ambrosia asymptomatic virus 1, **AobVX** *Adenium obesum* virus X, **APV1** Asian prunus virus 1, **ASPV** Apple stem pitting virus PA66, **BVX** Botrytis virus X NZL/Howitt. **CLBV** Citrus leaf blotch virus, **CsVX** Cassava virus X, **ICRSV** Indian citrus ringspot virus K1, **LOLV** Lolium latent virus, **PVM** Potato virus M (strain Russian), **PVX** Potato virus X, **ShVX** shallot virus X.
